# Prospective multicentre randomized double-blind placebo-controlled parallel group study on the efficacy and tolerability of StroVac® in patients with recurrent symptomatic uncomplicated bacterial urinary tract infections

**DOI:** 10.1007/s11255-022-03379-y

**Published:** 2022-10-01

**Authors:** S. Nestler, C. Peschel, A. H. Horstmann, W. Vahlensieck, W. Fabry, A. Neisius

**Affiliations:** 1 Urogate, Urological Practice, Bad Vilbel, Germany; 2 Strathmann GmbH & Co. KG, Hamburg, Germany; 3 Department of Urology, Kurpark Klinik, Bad Nauheim, Germany; 4 TFP Laboratory Düsseldorf, Düsseldorf, Germany; 5 Department of Urology, Brüderkrankenhaus Trier, University of Mainz, Mainz, Germany

**Keywords:** Urinary tract infection (UTI), Recurrent UTI (rUTI), Vaccination, Non-antibiotic prophylaxis

## Abstract

**Purpose:**

To evaluate efficacy and safety of vaccination with StroVac compared to placebo in patients with recurrent urinary tract infections (rUTI).

**Material and methods:**

We performed a prospective, double-blinded, placebo-controlled study in patients with uncomplicated rUTI. Patients received three single intramuscular injections with StroVac every two weeks. Primary endpoint was the number of bacterial urinary tract infections (UTI) over 13.5 months after randomization and adjusted by the respective “baseline” value when comparing verum and placebo group. Secondary endpoints were the number of patients with non-recurrence, time to first recurrence, frequency of recurrences, and patients' self-assessment of quality of life using a validated questionnaire.

**Results:**

376 patients were randomized to both groups between January 2012 and March 2015. Mean age was 44.4 years. Patients were mainly female (98.4%). In the StroVac group (*n* = 188), the number of UTIs was reduced from 5.5 to 1.2, in the placebo group (*n* = 188) from 5.4 to 1.3 (*p* = 0.63). In patients with ≥ 7 UTIs prior to study inclusion, StroVac was statistically significantly superior to placebo (*p* = 0.048). However, in all other secondary endpoints, no statistical differences between the two groups could be seen (all *p* > 0.3).

**Conclusion:**

StroVac reduced the number of clinically relevant UTIs like in former studies but did not show statistically significant better results than the chosen placebo. Most likely, that was due to a, since confirmed, prophylactic effect of the chosen placebo itself. Therefore, placebo-controlled and double-blinded studies using a different ineffective placebo preparation are needed to determine the importance of StroVac in prophylaxis of rUTI.

## Introduction

Urinary tract infections (UTI) are a common disease affecting 11% of women worldwide at least once per year [1]. Of them 34–53% are treated for recurrent UTIs (rUTI) [2] defined as minimum 2 UTIs in 6 or 3 UTIs in 12 months [3]. The uropathogenic bacterial spectrum consists of *Escherichia coli* being responsible for up to 85% of UTIs, and *Staphylococcus saprophyticus*, *Enterococcus spp., Klebsiella pneumoniae,* and *Proteus mirabilis* together being responsible for the most of the remaining 5–15% [4, 5]. Besides behavioural modifications [3, 6], low–dose, long-term antibiotic prophylaxis has been the primary procedure for decades [6, 7]. But in most current guidelines antibiotics are no longer recommended as first-line prophylaxis [3, 10] due to increasing antibiotic resistance rates [3, 6] and because scepticism in the population towards long-term ingestion of antibiotics due to high rates of adverse events leads to low compliance and effectiveness [3, 6, 8, 9]. Therefore, non-antibiotic modalities such as OM-89 or D-Mannose with an intake regimen similar to long-term antibiotics have emerged and proven their efficacy in well-designed studies [8, 11–13]. Nevertheless, these products are far from ideal, often also showing low patient compliance, since the medication has to be taken on a daily basis for three months or even without a time limit [11–13].

StroVac offers a different approach consisting of 10 strains of inactivated uropathogens: six of *E.coli with* different serotypes and one of *K. pneumoniae*, *P. mirabilis*, *Morganella morganii* and *Enterococcus faecalis* each. Therefore, StroVac covers a wide range of uropathogens [14]. Furthermore, only three injections are required at intervals of 1 or 2 weeks followed by a single booster injection 12 months after the initial vaccination [14]. So this prophylaxis requires less patient compliance. Studies have proven the efficacy of intramuscular [14–16] as well as vaginal use [17, 18] during 6 months follow-up. Most recently, a 2 years follow-up study showed non-inferiority to a 3 months prophylaxis with nitrofurantoin in the first year and a significant advantage in the second year, if the booster vaccination was applied after one year [19]. Therefore, recent meta-analyses [20, 21] as well as most guidelines [3, 10] recommend non-antibiotic prophylaxis in general and D-Mannose, OM-89 and StroVac especially, but nevertheless still see a “burning” [22] need for further research.

Therefore, we conducted a prospective, multicentre, randomized, double-blinded and placebo-controlled study on efficacy and tolerability of StroVac in patients with rUTI.

## Patients and methods

This double-blinded, randomized and placebo-controlled phase IV study was conducted from January 2012 to March 2015 in 40 sites in Germany. This study was conducted in compliance with the ICH Guideline E6 (R1) on Good Clinical Practice and registered under EudraCT no. 2010-020882-25. Several ethic committees were involved, including a leading committee in Berlin and local ethic committees in regions with participating centres.

### Study participants

Patients of both sexes aged 18–80 years were included if they had at least five symptomatic uncomplicated bacterial UTIs within 12 months prior to study inclusion. In accordance with current literature, UTI diagnosis required a positive urine culture with ≥ 10^4^ CFU/ml and at least two clinical symptoms [10]. Patients were evaluated retrospectively at time of study inclusion for the number of UTIs, which were documented in patient’s history in the recruiting clinic or practice. Participating patients had to sign the informed consent form, and those presenting with complete data sets were accounted for analysis.

Exclusion criteria comprised complicated UTI, lower urinary tract symptoms due to other reasons than UTI, neurogenic bladder dysfunction, bladder or kidney stones, residual urine, pregnancy, acute systemic infectious disease, a malignancy in the recent 5 years or radiation therapy of the abdomen (without time limit) or any other body part within the last 5 years, malfunction of immunity due to concomitant diseases such as diabetes mellitus with unstable metabolic status, liver or renal insufficiency, or complications caused by presently used medications. Further exclusion criteria were therapy with StroVac any time prior study inclusion, therapy with antineoplastic agents five years prior study inclusion, therapy with Uro-Vaxom 3 years, instillation therapy 18 months and continuous antibiotic treatment (> 30 days) or postcoital antibiotic treatment six months prior study inclusion. During the time of this study in 2012–2015, D-mannose was not used as prophylactic therapy of rUTI and therefore not permitted. During the study period, concomitant therapy with the above mentioned substances was permitted as well, only antibiotics in case of a necessary treatment of a UTI were allowed.

### Study design

Patients were randomized 1:1 to prophylaxis with StroVac or placebo. Randomization was carried out online by an Interactive Voice Response System (IVRS). Female patients were stratified by menopausal condition (pre- vs. postmenopausal); in cases of prior hysterectomy, women aged up to 45 years were stratified in the premenopausal group and patients over 45 years in the postmenopausal group. All male patients were assigned to the premenopausal group. Subgroup analyses were performed for hormonal status (pre- vs postmenopausal), age, and number of UTIs prior to study enrolment.

All patients received three single intramuscular injections separated by an interval of two weeks ± 7 days. Patients were seen seven times during the study period on scheduled visits and interviewed by telephone twice (see Table 1). Further, unscheduled visits were strongly recommended when a patient suspected a UTI and also possible at any time if requested by patients or urologists for other reasons. The recommendation was repeated at every scheduled visit and was written on every side of the patients’ diary.

**Table 1:** Time points, mode and content of visits during the study on long-term prophylaxis with StroVac or placebo. Unscheduled visits were possible any time at patient`s or urologist`s request

Study visits protocol			
V 1	Day -14 to -1	Visit	Screening and baseline assessment
Treatment period			
V 2	Day 1	Visit	Randomization, first immunization
V 3	Day 15 ± 7	Visit	Second immunization, efficacy and safety
V 4	Day 29 ± 7	Visit	Third immunization, efficacy and safety assessments
V 5	Day 43 ± 7	Visit	Efficacy and safety assessments
Observation period			
V 6	Month 4 ± 14 days	Telephone call	Efficacy and safety assessments
V 7	Month 7.5 ± 14 days	Visit	Efficacy and safety assessments
V 8	Month 11 ± 14 days	Telephone call	Efficacy and safety assessments
V 9	Month 13.5 ± 14 days	Final visit	Efficacy and safety assessments

*V1–9* visit 1–9

### Endpoints

The primary endpoint was the number of bacterial UTIs over 13.5 months after randomization adjusted by the respective “baseline” value (ITT-population) when comparing verum and placebo group. Baseline adjustment was done by including the respective baseline value as covariate into the statistical model. Secondary endpoints were defined as the number of UTIs within 12 months observation period (visits V5–V9), number of patients with non-recurrence during the entire study period, time to first recurrence, and frequency of recurrences during the first 6 months vs. months 7–12 after finalisation of the immunization scheme. Furthermore, patients' self-assessment and quality of life were measured using a validated questionnaire compiled from EUROHIS-QOL-Score [23], GBB 24 scale [24] and further validated questions concerning the burden of symptoms [25]. The results at study end (V9) were compared to the results at baseline (Visit 1) and at the end of the treatment period (Visit 5).

### Study medication

The investigational medicinal products consisted of a basic suspension and a dry substance for preparing the suspension for injection. Finished verum suspension for one injection contained at least 10^9^ inactivated bacteria including, *E. coli* 7.5 × 10^8^, *M. morganii* 3.75 × 10^7^, *P. mirabilis* 3.75 × 10^7^, *K. pneumoniae* 1.5 × 10^8^, and *E. faecalis* 2.5 × 10^7^. Furthermore, the medication included the excipients sucrose/dextran, disodium hydrogen phosphate, potassium dihydrogen phosphate, sodium chloride, thiomersal (merthiolate), aluminium phosphate and phenol (in traces), and water for injection. The verum medication matched with the commercial product StroVac according to the Summary of Product Characteristics, version 2021 [26]. The placebo suspension contained the ingredients of the verum except bacteria, phenol and thiomersal.

### Statistical analysis

The sample size calculation was based upon an average of 6.2 UTIs in the year pre-study, a 50% placebo response and an additional 15% StroVac effect in reducing UTIs during the study period. The number of 6.2 UTIs was calculated from data of an earlier study [13] and adjusted to the conditions of our study. No preselection was carried out. For a power of at least 80%, these assumptions resulted in a sample size of 185 patients per group (= 370 patients to be randomized in total). The sample size calculation was performed using the program StudySize 1.09. (CREOSTAT HB, Enbarsvagen 11, 42655 V.Frolunda, Sweden).

The efficacy analyses were performed using the intention to treat (ITT) population. The safety evaluable population (SEP) was used for safety analysis. The primary endpoint was evaluated in an approach for testing the superiority of StroVac over placebo. The statistical test was performed using the generalized linear model. The number of recurrences was analysed analogously, the time until first recurrence was calculated using Kaplan–Meier life-tables. The difference between the treatment groups was analyzed by the log-rank test. Statistical analyses were carried out by Pharmalog– Institut für Klinische Forschung GmbH, München using SAS-System version 9.2, SAS Institute Inc.

## Results

Of the 412 patients screened for eligibility, 376 patients were randomized equally to both groups between January 2012 and March 2015 (i.e. 188 patients per group, see Table 2). 21 and 18 patients in the StroVac and placebo group, respectively, terminated the study prematurely for different reasons. In the verum group, 7 patients terminated the study after the first or second injection, 2 of them due to adverse events. In the placebo group, 1 patient terminated during injection phase, not due to adverse events. All other patients left the study for different reasons during the observation phase. Mean age was 44.4 years with a range from 18 to 80 years. There were no statistically significant differences between the groups in demographic data and mean UTI frequency in the 12 months prior enrolment (see Table 2).

**Table 2:** Characteristics of patients with rUTI under prophylaxis with StroVac or placebo

Patient characteristics				
	StroVac	Placebo	Total	*p* value
Number, *n* (%)	188 (50)	188 (50)	376 (100)	
Age (years), mean (SD)	43.5 (18.9)	45.3 (18.0)	44.4 (18.5)	0.345
Caucasian, *n* (%)	185 (98.4)	187 (99.5)	372 (98.9)	0.749
Females, *n* (%)	187 (99.4)	188 (100)	375 (99.7)	0.685
Premenopausal, *n* (%)	114 (61.0)	114 (60.6)	228 (60.8)	0.949
Postmenopausal, *n* (%)	73 (39.0)	74 (39.4)	147 (39.2)	

The Results are presented as: mean and standard deviation (SD) and number and percent [*n* (%)]

**Fig. 1:**
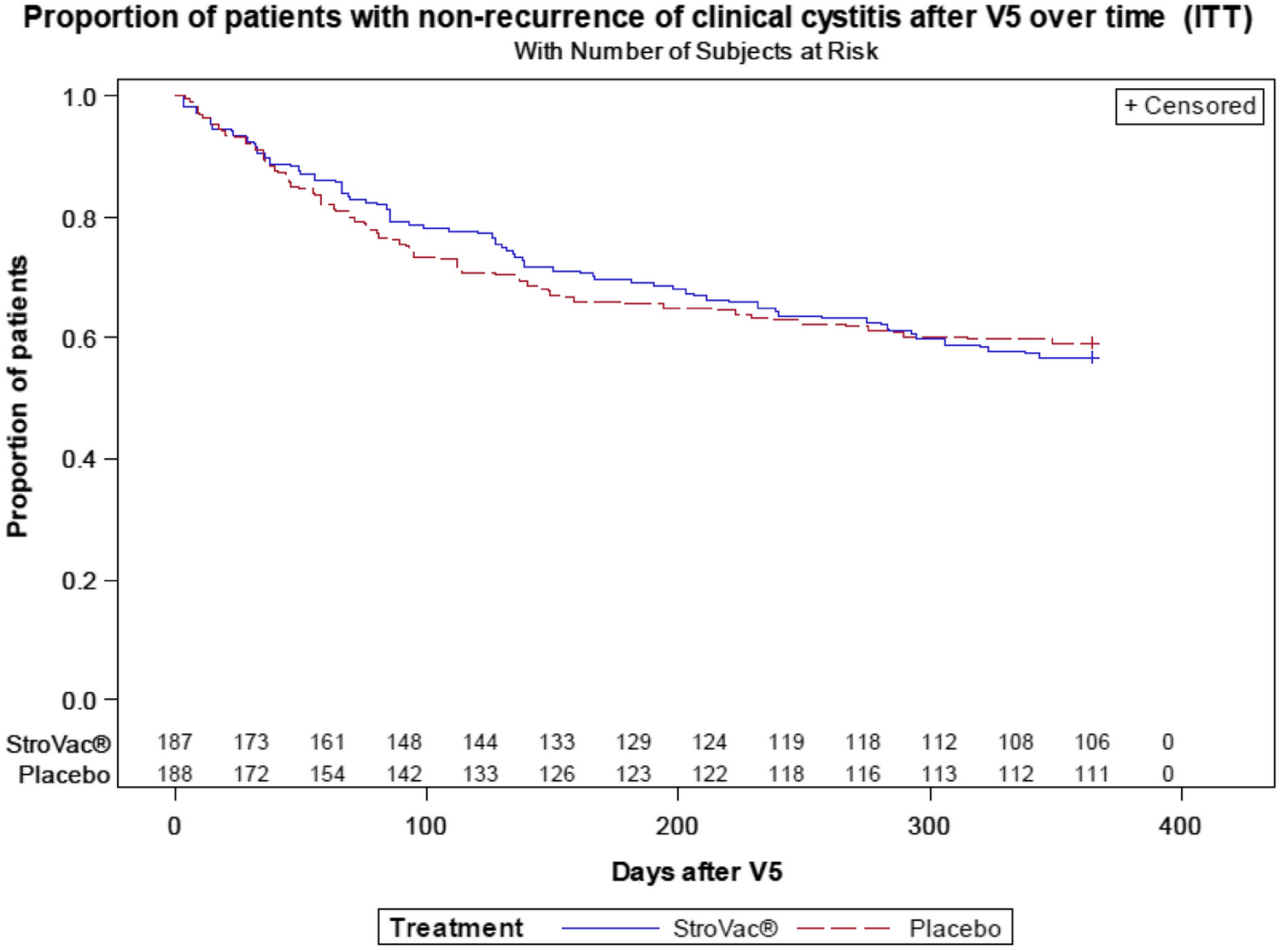
Patients with non-recurrence after visit 5 in StroVac and placebo group. 86 of 188 patients after StroVac (46.0%) and 97 of 188 patients (51.6%) after placebo experienced no recurrence twelve months after study inclusion (*p* = 0.33)

In the StroVac group, confirmed UTIs were reduced from an average of 5.5 within 12 months prior to study enrolment to 1.2 UTIs in the 13.5 months following first injection. Apparently, the placebo group showed similar results with a reduction from 5.4 UTIs to 1.3 UTIs. Therefore, a statistically significant difference in the primary endpoint (UTI recurrences) was clearly missed (*p* = 0.63, see Table 3). During the medically relevant 12 months observation period (visits V5–V9) similar results were seen with 1.0 and 1.1 UTI per year in the StroVac and the placebo group, respectively (secondary endpoint, *p* = 0.69). As well, no statistically significant difference could be shown regarding the time until first recurrence, which was 323 days in the StroVac group and 365 days in placebo group (*p* = 0.3). Overall, 183 patients had no UTI during 12 months following therapy, 86 of 188 patients after StroVac (46.0%) and 97 of 188 patients (51.6%) after placebo (*p* = 0.33, Fig. 1). The mainly detected bacterial species at the recurrences was *E.coli* (64.7% in both groups), followed by *Enterococcus spp* (7.7% in the StroVac group and 12.8% in the placebo group), *Klebsiella spp* (7.7 and 8.4%) and *Proteus spp* (1 and 3.1%). Other species did not exceed the proportion of 1% among the bacterial pathogens detected at recurrences. Surprisingly, in the StroVac patients, *S. aureus* followed *E.coli* as the second frequent species being responsible for 9.2% of UTIs in this group. Since *S. aureus* does not belong to the bacteria accounting for most UTIs [4] and its presence significantly differed between the groups, an additional analysis was performed without the patients with UTIs caused by *S. aureus*. Now, after StroVac vaccination and placebo, 50 and 51.6% of patients, respectively, were without recurrence; a still not significant difference [*p* = 0.3].

**Table 3:** Summary of results

	StroVac (*n* = 188)	Placebo (*n* = 188)	*p* value
Difference in the number of UTIs from baseline 12 months prior to 13.5 months after first injection	– 4.3 (5.5–1.2)	– 4.1 (5.4–1.3)	0.63
Time until first recurrence (days)	323	365	0.3
No UTI in the 12 months following therapy	46%	51.6%	0.33

Used for calculation was the analysis set ITT

### Subgroup analysis

In premenopausal women, no statistically significant difference was seen between the two treatment groups in the frequency of recurrences (*p* = 0.77), time until first recurrence (356.5 vs 358.5 days, *p* = 0.99), or percentage of patients with no UTI during follow-up (49.1 vs 50.0%, *p* = 0.76). Similar data were seen in postmenopausal women with no significant difference for recurrences (*p* = 0.81), time until first recurrence (239 vs 365 days, *p* = 0.14) or no UTI during follow-up (41.1 vs 54.1%, *p* = 0.78).

In patients older than 70 years, the reduction of frequency of UTI (by 4.4–4.5 UTIs, *p* = 0.44) and time until first recurrence (239 vs 365 days, *p* = 0.06) did not differ between the groups. However, the small number of patients (15 in StroVac group and 16 in placebo group) has to be considered.

Using a broader UTI definition (patients with self-documented UTIs without visiting a doctor, with monosymptomatic bacterial urinary tract infections, or with clinical cystitis without positive pathogen detection), a post-hoc subgroup of ITT patients who had more than 7 UTIs in the 12 months prior to randomization was analyzed. In this subpopulation (*n* = 58), a statistically significant difference could be demonstrated with a reduction of UTIs from 7.3 to 2.3 in verum patients compared to 7.6–4.4 in placebo patients (*p* = 0.048) after the follow-up of 13.5 months.

### Patient-reported outcomes/quality of life questionnaires (QoL)

In the StroVac group, complaints recorded in most QoL questionnaires were reduced by approximately 50% from V1 (baseline) to V9, in parallel to the reduction of UTI frequency. The mean scores of “symptom burden”, "mental consequences", and "effect on daily life" declined from 21.2 to 10.2, from 11.8 to 5.9, and from 9.7 to 4.4 points, respectively. But as the reduction in UTI frequency was similar in both treatment groups, no significant differences were observed in QoL as well. Even though statistical significance was nearly reached in the “mental consequences" score (*p* = 0.07), the p-values for the differences in all other scores were all above 0.34.

### Safety results

Overall, 619 adverse events (AE) occurred within the treatment period, 426 AEs in the StroVac group in 115 patients (62.2%), and 193 AEs in 83 patients in the placebo group (44.1%). By the end of the study, most AEs had resolved (98.7%). Most AEs were mild (54.9%) and moderate (33.4%) in intensity, and 11.6% were classified as severe. Most severe AEs occurred in the StroVac group (55 vs 17 AEs). Most common AEs were vaccination site pain (37.2% in the StroVac group and 5.3% in the placebo group) and influenza-like illness (11.7 and 4.8%, respectively). Most common severe AEs were vaccination site pain, pyrexia and influenza-like illness as well. 63.3% of patients with AEs reported in the StroVac group, and 17% of patients in the placebo group were classified as having drug-related AEs. 88.6% of the patients assessed the tolerability of StroVac as “very good” and “good”.

In the observation period after drug administration, 471 AEs occurred, equally in both groups showing 227 AEs in 81 patients in the StroVac group, and 244 AEs in 84 placebo patients. Again, most AEs were mild or moderate, severe AEs were more common in the placebo group. The most commonly reported event was nasopharyngitis (4.8 and 4.3% of all AEs in the StroVac and placebo group, respectively) followed by influenza-like illness (5.3 and 3.7%, respectively). Of all AEs, 2.7% were classified as drug-related in the StroVac group and 1.1% in the placebo group. Again, the majority of AEs had resolved by the end of the study.

## Discussion

Due to the emerging possibilities in non-antibiotic prophylaxis and a growing body of evidence proving their efficacy [8, 11–13, 27], current guidelines [3, 10] have changed, banning low-dose antibiotic prophylaxis from first-line treatment and instead promoting non-antibiotic prophylaxis. Nevertheless, there is still a need to evaluate non-antibiotic prophylaxis in long-term and double-blinded, placebo-controlled studies [22]. Therefore, we present the first GCP conform randomized, placebo-controlled study, evaluating the efficacy and safety of rUTI prophylaxis with StroVac.

Since the aim of the study was to prove the efficacy of StroVac in prophylaxis of rUTI, one has to state, that our study showed no significant advantage of StroVac over the placebo we used. We even saw a longer time till first recurrence and a higher rate of women with no UTI in the placebo group, even though not reaching statistical significance. But in spite of the failed statistical significance, StroVac reduced rUTI in patients with at least 5 UTI per year prior to prophylaxis to 1.0 UTI during follow up of 12 months. Overall, 71.7% of StroVac patients had only one or no UTI in the 12 months following the prophylactic injections.

These results are in accordance with the existing literature. In the largest non-interventional trial on StroVac published until today with over 1200 patients, the authors found a reduction from 3.5 UTIs in 6 months before to 0.6 UTIs in the 6 months following therapy [16]. Similar results were described in the most recent study by Nestler et al. [19], where 86.8% of patients had none or one UTI in the 12 months following vaccination compared to a median of 4 UTIs per year before therapy. This study was evaluated against Nitrofurantoin 100 mg once daily for three months and interpreted as successful since no statistically significant differences in the rUTI rates were seen [19]. Even though these studies had different inclusion criteria compared to our study and were according to the current definition of rUTI (3 UTI in 12 months or 2 UTI in 6 months), they showed similar results, so that we think, results are comparable.

When looking at other non-antibiotic prophylaxis regimens, similar results are described. Though, Wagenlehner et al. [27] reported a non-significant difference between OM-89 and placebo, most likely due to the small overall number of UTI in the observation period. Beerepoot and Geerlings [20], in a recent review, described the number of UTIs in patients treated with OM-89 as halved compared to placebo. One important study investigating D-Mannose prophylaxis in rUTI [8] found no recurrence in 85% of patients treated with D-Mannose and 80% of patients treated with Nitrofurantoin 50 mg during 6-month follow-up. Both studies showed a significant advantage compared to placebo, but did not surpass our results. Hence our study demonstrated an efficacy of StroVac that is comparable to its effects seen in former studies [14–19] and to the effects achieved with other non-antibiotic prophylaxis medication for rUTI [8, 11–13]. Head to Head studies comparing StroVac with other non-antibiotic prophylaxis substances are missing.

This placebo-controlled study confirmed that vaccination with StroVac leads to the expected effect in reducing rUTI frequency clinically relevantly but not statistically significantly better than placebo. The flaw in our study was the placebo preparation we used. Because the placebo achieved a 1.5 times higher effect than was expected, the preparation utilized in our study was further investigated for possible beneficial qualities. Indeed, the analysis revealed an antibacterial effect of the placebo itself [28]. Therefore, the placebo preparation used in our study was patented in 2019 under the name of dextran (patent no WO 2019/011514 A1). The effect on the bladder is most likely mediated by an activation of the immune system through the activation of macrophages, natural-killer cells and T- and B-cells [28]. Further investigations of the mechanisms are currently performed. Dextran might constitute another powerful non-antibiotic prophylaxis for rUTI in the future. Nevertheless, based on our results further studies are needed. StroVac as well as dextran both need to be tested in randomized double-blinded studies against a placebo preparation without antibacterial effect to determine their value and to show statistically significant differences.

Furthermore, the question remains, which definition of rUTI should be used in future studies. Since the study was conducted mainly in Germany, we used the definition provided by the German guidelines for recurrent uncomplicated urinary tract infections as established in 2010 [29] and renewed in 2017 [30]. But when applying the more “pragmatic” UTI definition according to the EAU guideline [3], our data show a statistically significant advantage of StroVac compared to our placebo for UTI patients who had suffered from ≥ 7 UTIs in the year prestudy. Considering these differences, tools for self-assessment should be part of the documentation in future studies, for example the Acute Cystitis Symptoms Score in any study on rUTI as suggested in the current German guidelines [10].

Lastly, since using heat activated bacterial strains, it is worth mentioning that we had only mild rates of reactions such as fever and flu-like illness, but despite the rate of 62.2% of AE in the StroVac group, 88.6% of the patients assessed the tolerability of StroVac as “very good” and “good”. This shows good tolerability and acceptance of the vaccine in patients. Even though comparable vaccines like whole cell pertussis vaccine showed high rates of AE, tolerability and safety in our study is supported by large studies on StroVac in Germany showing similar results [16].

Nevertheless, the obvious remaining limitation of our study is the failure to show a statistically significant superiority over the placebo effect. On the other hand, we believe that our work was a well-designed randomized and double-blinded trial with a large and well-balanced patient cohort. Thus, we consider our study an important contribution to research on non-antibiotic prophylaxis of rUTI providing confirmation of the clinically relevant prophylactic effects of StroVac in rUTI.

## Conclusion

Even though statistical significance of reducing recurrences in rUTI with StroVac in comparison to our chosen placebo was clearly missed, StroVac did reduce the number of rUTI clinically as expected from former studies. Nevertheless, so did the placebo and therefore we could not show a statistically advantage of StroVac over the chosen placebo with its antibacterial effect. Further randomized, placebo-controlled and double-blinded studies using a different ineffective placebo preparation are needed in the future to determine the importance of StroVac in prophylaxis of rUTI.
